# A Systematic Review of Artificial Intelligence‐Based Clinical Decision Support Systems in Prostate Cancer Management

**DOI:** 10.1049/htl2.70026

**Published:** 2025-11-18

**Authors:** Senobar Naderian, Farzin Soleimanzadeh, Leila Nikniaz, Sarvin Sanaie, Fatemeh Sadeghi‐Ghyassi, Taha Samad‐Soltani

**Affiliations:** ^1^ Department of Health Information Technology School of Management and Medical Informatics Tabriz University of Medical Sciences Tabriz Iran; ^2^ Cancer Artificial Intelligence Collaborative Group (CAICG) Universal Scientific Education and Research Network (USERN) Tabriz Iran; ^3^ Urology Department Tabriz University of Medical Sciences Tabriz Iran; ^4^ Tabriz Health Services Management Research Center Tabriz University of Medical Sciences Tabriz Iran; ^5^ Research Center For Integrative Medicine in Aging Aging Research Institute Tabriz University of Medical Sciences Tabriz Iran; ^6^ Research Center for Evidence‐Based Medicine, Iranian EBM Centre: JBI Centre of Excellence, Faculty of Medicine Tabriz University of Medical Sciences Tabriz Iran

**Keywords:** AI, CDSS, Gleason score, mp‐MRI, PCa

## Abstract

**Background:**

Prostate cancer (PCa) affects over 1.4 million individuals annually and remains one of the leading causes of cancer‐related death globally. Artificial intelligence‐based clinical decision support systems (AI‐CDSS) are increasingly used to improve diagnostic and treatment decisions in PCa management.

**Objective:**

This systematic review aims to evaluate the development, implementation, and limitations of AI‐based CDSS applied to the diagnosing, staging, treatment, or management of PCa.

**Method:**

The review protocol was registered with PROSPERO (ID = CRD42024498335) and adhered to preferred reporting items for systematic reviews and meta‐analyses (PRISMA) guidelines. Using the (population/intervention/comparison/outcome) PICO framework, research questions were formulated to examine system development, AI models applied, adherence to CDSS standards, and integration into clinical decision‐making phases. A systematic search of PubMed, Scopus, Web of Science and Embase retrieved 1137 articles; after removing 173 duplicates, 964 records were screened. A subsequent update search in June 2024 identified no additional papers. Two reviewers independently screened titles and abstracts, full texts, and resolved disagreements through discussion with a third reviewer. Studies were included if they developed and evaluated AI‐based CDSS for PCa with reported performance metrics. Studies were excluded if they did not involve AI‐based CDSS, lacked evaluation data, or did not address clinical decision‐making.

**Results:**

Ten studies met the inclusion criteria. Cohorts ranged from 10 to 1.93 million records. Reported tasks included diagnosis, risk/recurrence prediction, survival estimation, and radiotherapy/ brachytherapy plan optimization. Common models were gradient boosting, neural networks, and deep reinforcement learning (DRL). Performance was variable but modern ML generally outperformed traditional baselines for survival and risk prediction (e.g., AUROC ≈ 0.84 for three‐year recurrence; C‐index up to 0.92 for five‐year progression‐free survival (PFS)). A workflow CDSS without predictive modeling reduced decision‐making time by 60%. Key limitations included small or heterogeneous samples, limited generalizability, inconsistent or incomplete data inputs, scarce external validation/calibration, and interpretability concerns. Core CDSS components were inconsistently implemented.

**Conclusions:**

AI‐based CDSS for PCa show promise for diagnosis, prognosis, and treatment planning and may improve efficiency. However, broader adoption will require high‐quality, multi‐site data; external validation and calibration; interpretable models; and robust integration with EHRs and clinical workflows. Prospective clinical evaluations are needed to confirm patient‐level impact.

## Graphical abstract

This systematic review investigates AI‐CDSS for PCa. It examines data inputs, AI models, system integration, and clinical application, highlighting limitations and implementation gaps. Findings underscore the need for more comprehensive, interoperable, and validated AI‐CDSS for real‐world use.

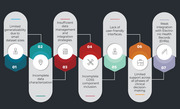

## 1 Introduction

PCa accounted for approximately 1.47 million new cases and about 397,000 deaths worldwide in 2022 [1]. It was the second most frequently diagnosed cancer in men, with incidence varying across countries; this emphasizes the need for early detection, effective treatment, and public awareness [2]. Despite these efforts, challenges persist in diagnosis, treatment, and follow‐up [2]. Current screening methods, particularly the prostate‐specific antigen (PSA) test, are hindered by low specificity and a risk of overdiagnosis, making early detection challenging [2]. Tumor heterogeneity and aggressiveness complicate both risk assessment and treatment selection, requiring careful balancing among radiation, surgery, hormonal therapy, and active surveillance [2, 3]. Although targeted therapies and immunotherapies show promise, they require further integration and validation [3]. Effective follow‐up, supported by clinical imaging and genetic data, is essential for detecting recurrence and guiding treatment decisions [4, 5].

Clinical decision‐making in PCa typically involves three phases: diagnosis, severity assessment, and management [6]. Diagnosis involves identifying the disease based on patient data [6]; severity assessment evaluates the stage and risk of complications [7]; and management entails selecting an appropriate treatment plan tailored to the patient [8].

**FIGURE 1:**
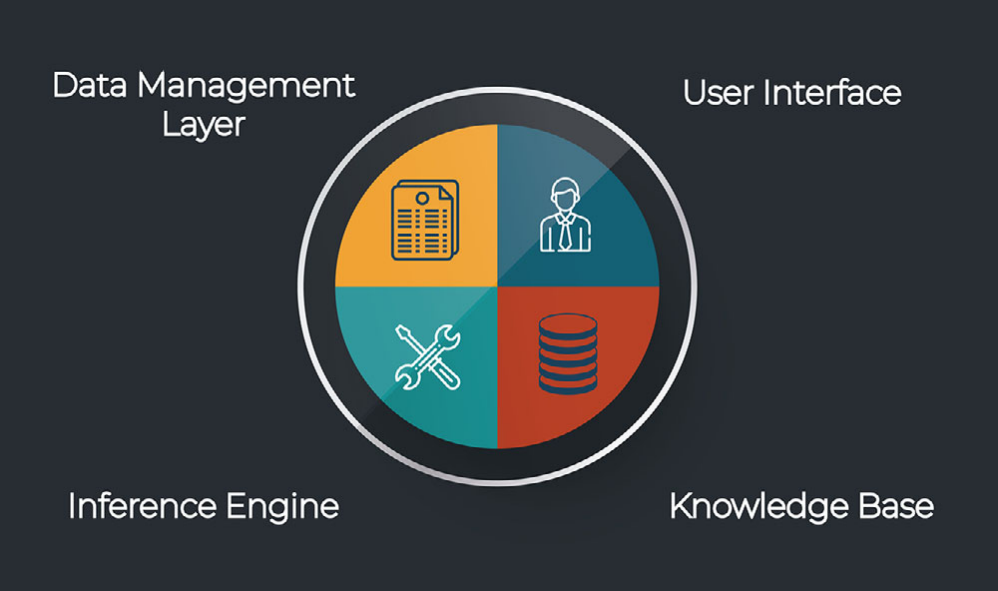
A simple breakdown of the core components of CDSSs.

A CDSS assists clinicians in one or more phases of decision‐making by providing evidence‐based recommendations tailored to patient data [9]. CDSSs are commonly classified as knowledge‐based or data driven (non‐knowledge‐based) systems [10]. Knowledge‐based CDSSs use rule‐based inference engines linked to an explicit knowledge base [11]. Non‐knowledge‐based CDSSs, including AI‐based systems, rely on patient data and use statistical or AI‐models to forecast future outcomes or generate recommendations [10]. Essential components of AI‐driven CDSS include a knowledge base, an inference engine, a data management layer, and a user interface (UI) [9, 12, 13]. These components are illustrated in Figure 1, which outlines the typical architecture of an AI‐based CDSS.

The knowledge base uses clinical guidelines and patient data to generate recommendations, while the inference engine applies AI algorithms (like machine learning or natural language processing) to generate clinical suggestions [12]. The data management layer makes sure that the input data is processed reliably [12]. AI‐based CDSSs are rapidly growing, supporting healthcare providers in making more accurate decisions [14], and have already shown success in improving diagnostic and treatment accuracy for various medical conditions, including [15].

Research Questions:

1. What population and data characteristics (e.g., sample size, demographics, PSA levels, medical history, imaging) were used in CDSS development?
2. Which AI algorithms were implemented, what target variables were evaluated, and what performance metrics were reported?
3. Do the systems adhere to CDSS standards, including user interaction, integrating with electronic health record (EHR) and electronic medical record (EMR), or multi‐source data handling?
4. How did the systems align with the clinical decision‐making phases, such as diagnosis, staging, or treatment recommendations?

Therefore, this review aims to assess the development and application of AI‐based CDSSs in PCa management, focusing on the data and methods used, the AI models and reported performance metrics, adherence to CDSS design standards, and integration into clinical decision‐making phases.

## 2 Methods

Protocol and registration: This review was registered in PROSPERO (ID: CRD42024498335) and conducted in accordance with the PRISMA guidelines for systematic reviews [16].

The research questions were framed using the PICO format, as follows:

Population (P): Individuals at risk of PCa, those undergoing diagnosis, or patients with confirmed PCa.

Intervention (I): Development of AI‐based CDSSs for the diagnosis, classification, treatment, or management of PCa.

Comparison (C): Standard care or no intervention.

Outcome (O): Diagnostic accuracy, clinical relevance, or other metrics related to the development and performance of the CDSSc.

### 2.1 Eligibility Criteria

Inclusion criteria: Studies must focus on AI‐based CDSSs developed for PCa, using AI methods such as deep learning or ensemble methods. The system must support healthcare professionals in clinical decision‐making, specifically for diagnosis, assessment, or management. The target population should be PCa patients, including those undergoing diagnosis or management. Only primary research studies reporting AI performance metrics and CDSS‐related outcomes were included.

Exclusion criteria: Studies that do not involve AI‐based CDSS or focus solely on pre‐existing systems, studies that do not use AI methods (like traditional algorithms), or studies not published as primary research are excluded. Additionally, studies must report relevant performance outcomes or be available for analysis in English. Studies on non‐human subjects, duplicate publications or not focused on clinical decision‐making for PCa are excluded.

### 2.2 Information Sources and Search Strategies

A systematic search was conducted on 1 January, 2024, across four major databases: PubMed, Scopus, Web of Science, and Embase, yielding 1137 retrieved papers. After removing 173 duplicates, 964 papers were screened. Subsequently, an update search on 26 June, 2024, yielded no new relevant studies. Additional searches were done on Google Scholar, and both free‐text and controlled vocabularies were used in the search strategy.

The search strategy employed across databases, included a criterion, is available in Supplementary File 1. A full‐search strategy for PubMed is provided in Supplementary File 2.

### 2.3 Screening, Data Extraction, and Quality Assessment

The screening process was conducted by two reviewers (SN, TS) who independently assessed the titles, abstracts, and full texts of articles according to inclusion and exclusion criteria. Disagreements were resolved by discussion or by consulting with a third reviewer (FS). In the next step, two reviewers (SN, TS) assessed the whole text of articles separately and used standardized Microsoft Excel spreadsheets for data collection (i.e., citation, year, population, CDSS structure, purpose of design, AI methods, outcome measures, key findings/results, and limitations). The quality of the included studies was also independently assessed by two researchers (S, FS‐G). Given the specialized nature of system developmental studies, we assessed them based on a set of custom‐made criteria focusing on key areas: clarity in population description, the description and performance of AI algorithms, adherence to clinical decision support standards, and alignment of each CDSS with the clinical decision‐making, focusing on their application to the specific phases. Any disagreements were resolved by consensus.

### 2.4 Synthesis of Results

Due to the heterogeneity of study populations, interventions, and results, a meta‐analysis was not feasible. Therefore, a narrative synthesis was performed, focusing on:

- The population on which the CDSSs was developed, including sample size, demographics, and data factors (such as PSA levels, medical history, and imaging data).
- AI algorithms used, target variables, and reported performance metrics.
- The alignment of the system with clinical decision support standards, such as offering user interaction beyond data input and integrating with EHR, EMR, or handling data from multiple sources.
- The conformance of the CDSS workflow with clinical decision‐making phases (diagnosis, severity assessment, or treatment recommendations) targeted by the system.

See Supplementary Files 1‐3 for full search strategies, extraction headings, and the PRISMA 2020 checklist mapping.

## 3 Results

### 3.1 Selection of Sources of Evidence

**FIGURE 2:**
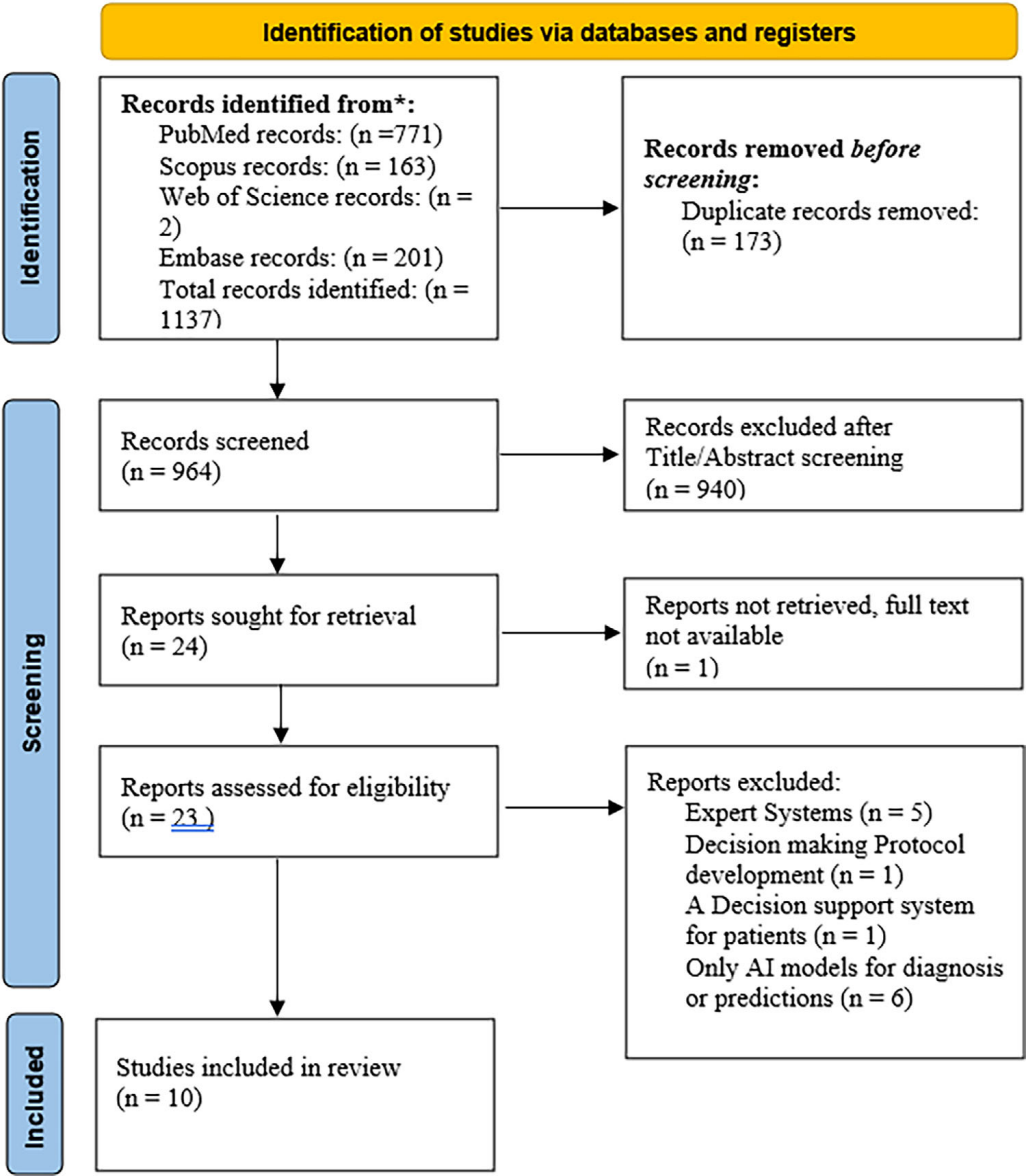
The PRISMA flow diagram.

After removing 173 duplicates from an initial total of 1137 retrieved papers, 964 unique records remained. After title/abstract screening, 940 records were excluded, leaving 24 full text articles for eligibility assessment. One article's full text could not be retrieved, despite contacting the authors. The remaining 23 papers were assessed in detail. After full‐text review, 13 articles were excluded: five were found to be expert systems [17], one developed a decision‐making protocol, one was a patient‐facing system, and six focused solely on AI models for diagnosis or prediction. Ultimately, 10 studies met the inclusion criteria. The PRISMA flow diagram (Figure 2) shows the selection process.

### 3.2 Characteristics of Sources of Evidence

The characteristics of the included studies are summarized in Table 1.

**TABLE 1:** Characteristics of included studies.

Row	Citation	Publication year	Patient population	Structure of CDSS	The purpose of design	AI methods	Outcome measures	Key findings/results	Limitations
1	[18]	2012	24 prostate cancer patients undergoing MRI and robot‐assisted radical prostatectomy (RARP). The average patient age of 60.4 years and a mean PSA of 6.62 ng/mL.	The CDSS integrates multiparametric MRI (mp‐MRI) data with histopathology using a Java graphical user interface (GUI) as a plug‐in tool for MIPAV image processing software, MATLAB, and RAPIDMINER software workflow.	To develop and evaluate a CDSS for prostate cancer diagnosis using mp‐MRI data for creating cancer probability maps and improving the accuracy of cancer diagnosis.	Support vector machines (SVMs) to classify regions of interest (ROIs) within prostate as cancerous or non‐cancerous. Genetic algorithm to optimize the parameters of SVM model. Unsupervised learning (model‐based expectation maximization (EM) algorithm) generate cluster maps from mp‐MRI features	Diagnostic accuracy: evaluated through leave‐one‐patient‐out cross‐validation (LOPOCV), comparing MRI findings with histopathological results. Efficacy of the system: using accuracy, sensitivity, specificity, positive predictive value (PPV), negative predictive value (NPV), and Kappa coefficient./ Balance between sensitivity and PPV: measured by F‐measures.	Significant differences (*p* < 0.0001) were found in all the measured mp‐MRI parameters T2‐weighted, apparent diffusion coefficient (ADC), T1, volume transfer constant (Ktrans), rate constant (kep) values between cancerous and noncancerous prostate regions based./ The SVM‐based CDSS achieved an F‐measure of 89% and a Kappa coefficient of 80% after SVM parameter optimization, showing an agreement with histopathology results.	Small sample size (24 patients)/ dependency on specific MRI sequences and parameters/ validation limited to RARP specimens, potentially limiting generalizability.
2	[19]	2020	7267 patients with biopsy‐confirmed prostate cancer from the Seoul cancer panel database (SCaP) with complete clinical data, standard treatments, with complete clinical data and follow up.	Web‐based decision support system (DSS) with a user interface (UI) for manual data input and visualization of results (patient's Kaplan‐Meier survival curve).	To develop and evaluate a CDSS for survival outcomes prediction in prostate cancer patients that is comparable to traditional methods of Cox proportional hazards model (Cox) proportional hazard model.	Multilayer perceptron (MLP), MLP for N‐year survival prediction (MLP‐N), long short‐term memory (LSTM). All models were three‐layer, feed‐forward with sigmoid activations.	Progression‐free survival (PFS) time from diagnosis to development of castration‐resistant prostate cancer (CRPC). Cancer specific mortality (CSM). Overall mortality(OM). Area under curve (AUC) to assess model ability to discriminate between patients who will and will not experience death, Harrell's C‐index a survival analysis metric that is akin to AUC but focuses on patient concordance when forecasting event order./ accuracy, sensitivity, specificity, positive and negative predictive values at a specific cut‐off value.	Artificial neural network (ANN) models, especially the LSTM model, outperformed the Cox‐regression model in predicting PFS, CSM and OM at 5 and 10 years. LSTM achieved a 92% C‐index for PFS predicting at 5‐year versus Cox's 89%./ LSTM showed superior accuracy, sensitivity (88%), specificity (83%), PPV (75%), and NPV (90%) compared to other models in predicting survival outcomes.	Limited to a Korean population; cultural biases in treatment choices; retrospective study design/ lacks recommendation / CDSS structure limitations include insufficient detail on decision‐making algorithms and integration with existing clinical workflows.
3	[20]	2020	5114 prostate cancer patients in South Korea.	Web‐based application with a prediction model trained on a national prostate cancer database.	Development of a CDSS for predicting biochemical recurrence (BCR) at three and five years post‐radical prostatectomy.	gradient boosting classifier (GBC).	AUC, accuracy, sensitivity, specificity, Matthews correlation coefficient (MCC)	GBC model showed highest performance with AUC of 0.8419 for 3‐year and MCC of 0.4836 for 5‐year predictions.	Limited to Korean population/ follow‐up period variability lacks recommendation.
4	[21]	2020	74 prostate cancer patients.	Includes an interface for user interaction, integration with a deep neural network named virtual treatment planner network (VTPN), and optimization engine.	To develop a system to automate and optimize treatment planning parameters (TPPs) in prostate cancer Intensity‐modulated radiation therapy (IMRT) in an end‐to‐end deep reinforcement learning (DRL) process.	Deep reinforcement learning (DRL), Q‐learning, neural networks.	Plan quality assessed, considering dose delivered to the prostate (PTV) and nearby organs like bladder and rectum, through original and modified ProKnow scores. Higher scores indicate better plans that spare organs at risk (OARs) and ensure proper target volume coverage. Time to complete the treatment planning process.	Significant improvement in plan quality scores with VTPN‐guided adjustments compared to initial plans; average scores increased from 4.84 to 8.45 (original ProKnow score) and from 6.07 to 10.93 (modified score).	Computational intensity during training and optimization phases. potential lack of interpretability of VTPN's decision‐making process. reliance on simplified metrics like dose‐volume histograms (DVHs) rather than 3D dose distributions.
5	[22]	2020	1,933,535 records aggregated across multiple institutions; mixture of confirmed PCa cases and broader administrative records as reported.	An auxiliary multi‐layer system based on multi‐institutional dataset, to process multiple patients' simultaneous diagnosis requests.	To develop a system to improve diagnostic accuracy, optimize treatment strategies, and enhance overall management of prostate cancer.	Support vector machines (SVM) for binary and multi‐class classification./ Neural networks (multilayer perceptron‐(MLP) and radial basis function‐(RBF) for classification. Exponential linear regression (ELR) for ensemble learning	System accuracy compared to physicians/ effectiveness of treatment plans.	The system's accuracy gets close to that of physicians when the data reaches 4000. A rough 0.5 deviation in evaluation of malignancy (EM) values around the supervising value set for different malignant samples.	Limited to initial implementation and testing data from specific hospitals in China. potential overfitting on the training dataset. not tested across diverse demographics.
6	[23]	2020	over 8,000 patients diagnosed with prostate cancer across three major hospitals in China	Integrated AI‐based system utilizing medical data using a multilayer neural network incorporating inputs from disease and medical image indicators.	To develop and evaluate a CDSS for intelligent prostate cancer diagnosis and to improve diagnostic accuracy compared to traditional methods.	Neural networks, including multilayer perceptron (MLP). Using data analysis techniques such as Preprocessing, Feature Extraction, Model Training and Decision‐Making.	Comparison of diagnostic accuracy between system and medical experts.	High initial diagnostic accuracy when number of cases was relatively low, with experts achieving up to 97% accuracy. As the number of cases increased from 200 to 8000, the diagnostic accuracy of human experts decreased from 97% to 81%. And the system's diagnostic accuracy improved from 61% to 87%. The true positive rate of the system was found to be at the optimal point (0,[1], indicating high diagnostic accuracy, according to the study of the receiver operating characteristic (ROC) curve.	Limited to specific demographic groups. Dependency on accurate and extensive data for optimal performance. variability in data quality across hospitals. real‐world applicability may vary.
7	[24]	2022	18,824 patients from Seoul National University Bundang Hospital (SNUBH) and SMG‐BMC.	Web‐based tool with a flask backend, accessible through a user‐friendly HTML5 interface.	To develop and validate a CDSS for predicting prostate cancer and clinically significant prostate cancer (CSPC) in patients with PSA levels 2.0‐10.0 ng/Ml.	Dense neural network (DNN), extreme gradient boosting (XGBoost)	area under the receiver operating characteristic curve (AUROC), comparison with traditional biomarkers and prostate health index (PHI).	DNN AUROC for PC: 0.743 (internal), 0.704 (external); outperformed traditional biomarkers; DNN significantly outperformed PHI in a subgroup of 259 patients.	Limited generalizability to other settings. Reliance on imputed missing data. Limited generalizability beyond the PSA gray zone due to study inclusion criteria and retrospective nature.
8	[25]	2022	10 PCa patients undergoing screening for diagnosis.	AI‐Pathway Companion: Integrates data from multiple hospital information systems (LIS, RIS, PACS, pathology) into a unified patient dashboard. Uses natural language processing and FHIR (Fast healthcare interoperability resources) standards.	To assist in decision‐making for prostate adenocarcinoma by providing diagnostic and therapeutic recommendations based on clinical guidelines, including data aggregation and pattern recognition.	NLP, fast‐healthcare Interoperability Resources (FHIR), AI for pattern recognition, data aggregation, and standardization.	59.9% reduction in decision‐making time(total case preparation time reduced from 191.44 to 76.72 s). Significant improvements in completeness, format, and understandability of patient data. High satisfaction ratings (Cohen's d = 4.94).	Single institution study limits generalizability. Only 10 readers were observed, limiting the ability to assess inter‐user variability. The study only evaluates pre‐therapeutic prostate cancer management, limiting the system's applicability in advanced stages.	The study was conducted at a single institution, which limits the generalizability of the findings to other healthcare settings or systems. The study involved only 10 clinicians, limiting the assessment of inter‐user variability. The software was only assessed for the pre‐therapeutic phase of prostate cancer management, and further studies are needed to evaluate its effectiveness in advanced cancer stages or post therapeutic management.
9	[26]	2023	14 prostate cancer patients undergoing single‐dose high‐dose rate (HDR) brachytherapy.	A system called BRIGHT, (A proprietary CDSS for HDR brachytherapy treatment scheduling) software, bi‐objective planning model, that considers two primary objectives: coverage of the prostate and seminal vesicles, and sparing of surrounding organs, EA MO‐RV‐GOMEA (evolutionary mlgorithm (EA), multi‐objective real‐valued gene‐pool optimal mixing evolutionary algorithm (MO‐RV‐GOMEA))	To develop a system that Optimizes HDR brachytherapy treatment planning. To provide radiation oncologists with a set of optimal plans that offer different coverage‐sparing trade‐offs. And to reduce treatment planning time and increase its quality.	Evolutionary algorithm (EA) called MO‐RV‐GOMEA, that optimizes the normalized least coverage index (LCI) and normalized least sparing index (LSI) functions to find plans that balance target coverage and organ‐at‐risk (OAR) sparing. Pareto optimization that finds a set of plans where improvement in one objective can only be achieved at the expense of other objectives.	Dose‐volume parameters, normalized LCI and LSI, plan approval time, plan selection time.	Optimized plans met dose‐volume criteria more effectively. reduced time spent on plan selection and approval. provided better trade‐offs between target coverage and organ‐at‐risk (OAR) sparing. Clinically approved plans required manual fine‐tuning in some cases. Time per process step was recorded, showing potential reductions in planning time if the import‐export steps were optimized.	Need for manual fine‐tuning in certain cases. limitations in achieving optimal plans due to suboptimal implant geometry. Additional training required for radiation therapy technologists (RTTs). Time‐consuming import‐export process between BRIGHT and Oncentra Brachy (OB). limited sample size with only 14 patients.
10	[27]	2023	801 patients with castration‐resistant prostate cancer (CRPC).	a web‐based decision support system (DSS) based on the final models, which integrates into EHR system, accessible for clinical implementation online.	To develop a machine learning based decision support system (DSS) to predict castration‐resistant prostate cancer (CRPC) patient survival and guide treatment selection for first‐line, second‐line, and third‐line therapies.	Supervised learning techniques such as Logistic Regression, Random Forests, and Support Vector Machines (SVM). Data preprocessing techniques including normalization and imputation of missing values. Cross‐validation and hyperparameter tuning to optimize model performance.	Primary Endpoints of Cancer‐Specific Mortality (CSM) and overall mortality (OM)./ Secondary endpoint of development of a decision support system (DSS) based on machine learning analytics.	Performance of Predictive Models: Extreme gradient boosting (XGB) models trained with non‐imputed datasets showed the best performance./ C‐index (Harrell's concordance index) for CSM models: First line: 0.827 (95% CI, 0.768–0.880). Second line: 0.807 (95% CI, 0.709–0.882). Third line: 0.748 (95% CI, 0.635–0.857). C‐index for OM models: First line: 0.822 (95% CI, 0.758–0.878). Second line: 0.813 (95% CI, 0.719–0.890). Third line: 0.729 (95% CI, 0.613–0.846). Identified important risk factors influencing outcomes (CSM and OM): Hemoglobin levels, PSA levels at various stages, and alkaline phosphatase were among the top factors. SHapley Additive exPlanations (SHAP) plots showed the distribution and impact of these factors on model predictions. Clinical Application.	Relies heavily on the quality and completeness of EHR data. Findings may be specific to the cohort studied and may not generalize to other populations or healthcare settings. Machine learning models used are often considered black‐box, limiting interpretability of decision‐making processes. Handling patient data raises concerns regarding privacy, security, and compliance with healthcare regulations.

*Note*: n = sample size; values and model details are summarized from the cited publications.

Abbreviations: CDSS, clinical decision support system; PCa, prostate cancer; GUI, graphical user interface; mp‐MRI, multiparametric MRI; PSA, prostate‐specific antigen; EMR/EHR, electronic medical/health record; DNN, deep neural network; LSTM, long short‐term memory; SVM, support vector machine; RBF, radial basis function; DRL, deep reinforcement learning; EA, evolutionary algorithm; DVH, dose‐volume histogram; PFS, progression‐free survival; CSM, cancer‐specific mortality; OM, overall mortality; BCR, biochemical recurrence; CSPC, clinically significant prostate cancer; NR, not reported.

## 4 Narrative synthesis of results

### 4.1 Addressing Research Question 1

Sample size: Sample sizes varied widely across studies. Three studies [18, 21, 26] had small sample sizes (10 to nearly 70 patients), focusing on developing or evaluating specific subsets of CDSS. Several studies [19, 20, 22, 23, 24] used larger cohorts (thousands to 1.9 million records) Notably, Song et al. [24] analysed 18,824 patients in the PSA gray zone; Zhang et al. [23] analysed over 8000 PCa diagnosed patients; and Wu et al. [22] reported approximately 1.93 million records aggregated from multiple institutions.

Data characterization: The data used to develop models varied significantly. Studies aiming for generalizability [19, 20, 22, 24] used a wider range of data points. Research focused on particular aspects such as diagnosis or treatment planning [18, 21, 26], often used specialized data sets. Study [25] integrated contextual clinical data, PSA, age, DRE, prostate volumes, prostate imaging reporting and data system (PI‐RADS) scores, and biopsy results from laboratory information system (LIS), radiology information system (RIS), picture archiving and communication system (PACS), and clinical information system (CIS) via a fast‐healthcare interoperability resources (FHIR)‐based connector. Data factors in the included studies can be categorized in the following aspects: Demographics and clinical data: Studies [19, 20, 22, 24, 27], included a range of clinical and demographic factors such as age, BMI, PSA levels, TNM (tumor, node, metastasis) stage, comorbidities and specifics of the treatments (such as ECOG score). Studies [18, 26], used limited data, like only age and basic treatment information [26] or PSA level [18]. Notably, study [21] focused solely on treatment plan data and omitted clinical variables. More generally, study [25] included contextual clinical information (prostate volume, age, DRE, and PSA) in a structured format aligned with SNOMED CT and LOINC. Biopsy data: Studies [18, 21, 22, 23, 24, 26], did not specifically address using biopsy data, while studies [19, 20] included pathological Gleason score, T stage, with study [27] also incorporating Gleason score. The software dashboard of study [25] included biopsy results and suggestions, though pathological staging information was missing. Imaging data: MRI and PET‐CT imaging were used in studies [25, 26] (pelvic T2‐weighted, diffusion‐weighted, DCE‐MRI sequences), whereas study [23] used PET‐CT data to analyse lesion area and density. Many studies [19, 20, 21, 22, 24, 27] did not address the use of imaging data. Other data points: Besides the fundamental categories, some other data were also included in studies. The treatment plan data with dosage volume histograms and weighting factors were the main focus of study [21]. Studies [24, 27], incorporated laboratory results other than PSA (e.g., hemoglobin, alkaline phosphatase (ALP), and fPSA). Study [20], used EMRs data. Studies [22, 24], however, noted missing data points for a few variables like fPSA, and transition zone volume (TZV). Also in study [25], manual data replacement was permitted in some circumstances when digital data was not available. Publicly accessible demonstration versions of the decision support calculators developed by Koo et al. [19], and Lim et al. [27] are available online.

### 4.2 Addressing Research Question 2

AI algorithms, targets, and performance metrics: The studies employed a range of AI algorithms used for PCa CDSS development. Optimization methods were used in studies [26] and [21], where study [26] employed a multi‐Objective Real‐valued gene‐pool optimal mixing algorithm for optimizing treatment plans, and study [21] used a VTPN based on DRL for optimizing intensity modulated radiotherapy (IMRT) plans. In study [19] (LSTM and MLP networks for survival prediction) and in [22] (SVM), MLP, RBF‐kernel models, and other neural networks for diagnosis, staging, and treatment), ANNs were investigated. Study [20] used GBC for BCR prediction, and study [27] used XGBoost for survival prediction and treatment selection; both used gradient boosting techniques. Furthermore, the Cox proportional‐hazards regression model was used for comparison in studies [27] and [24]. RBF‐kernel‐equipped SVMs were employed in studies [18] (mp‐MRI‐based categorization of cancerous versus non‐cancerous tissue) and [22](tumor diagnosis). Study [23] used a perceptron neural network model that combined imaging data for diagnosis. The target variables varied: Studies [26], optimization of the HDR brachytherapy plan, and [21], optimization of the IMRT plan, focused on treatment optimization. Studies [19] and [27] focused on predicting survival, whereas studies [20] and [24] attempted to predict the likelihood of BCR and the risk of CSPC, respectively. Studies [22] and [23] examined the diagnosis of PCa (malignant vs. benign), with study [22] also looking into disease stages and recommending treatments. Additionally, study [22] included treatment efficacy evaluation as a target. The target variable also had an impact on the performance metrics that were used to assess the CDSS models. Research on treatment plan optimization [21, 26] included variables such as planning time, plan quality scores (ProKnow), and dose‐volume parameters. Studies on survival prediction [19, 27] reported metrics including AUC, sensitivity, specificity, accuracy, and Harrell's C‐index. AUC, accuracy, sensitivity, specificity, and other metrics such as the MCC were used in BCR prediction studies [20, 24]. Metrics like accuracy, sensitivity, specificity, and AUC (ROC AUC) were used for diagnostic studies [18, 22, 23]. Some studies compared their models to validated biomarkers or Cox regression) for added reliability. Study [25] did not include predictive modeling; instead, it applied natural language processing and pattern recognition techniques to enhance clinical workflow, reducing decision‐making time by 59.9% and improving user satisfaction.

Table 2 summarizes the model families, clinical tasks, cohort sizes, and the primary performance metrics that authors reported (AUROC), Harrell's C‐index, accuracy, sensitivity, specificity, PPV/NPV, and MCC). Where studies evaluated operational outcomes instead of predictive accuracy (like plan‐quality scores, decision‐making time); we report those; NR denotes metrics not reported.

**TABLE 2:** Summary of AI‐CDSS performance and key outcomes across included studies.

Row	Citation	Publication Year	Clinical task	Model(s) evaluated	*n*	Primary metric(s) reported	Best reported value(s) / key outcome(s)
1	[18]	2012	Diagnosis/localization from mp‑MRI	SVM (with GA tuning)	24	F‑measure; κ; (Acc/Sens/Spec also reported)	F‑measure 0.89; κ 0.80
2	[19]	2020	Survival (PFS, CSM, OM)	LSTM; MLP/MLP‑N vs Cox	7,267	C‑index; Acc/Sens/Spec/PPV/NPV	LSTM C‑index 0.92 (5‑yr PFS) vs Cox 0.89; Sens 0.88; Spec 0.83; PPV 0.75; NPV 0.90
3	[20]	2020	Biochemical recurrence (3‑ and 5‑yr)	Gradient boosting classifier	5114	AUROC; MCC	AUROC 0.842 (3‑yr); MCC 0.484 (5‑yr)
4	[21]	2020	IMRT plan optimization	Deep RL (VTPN)	74	ProKnow plan‑quality score; planning time	ProKnow (orig) 4.84→8.45; (mod) 6.07→10.93; planning time ↓ (NR)
5	[22]	2020	Dx/staging/Tx recommendation	SVM; MLP; RBF; ELR (ensemble)	1,933,535	Accuracy vs physicians; EM deviation	“Accuracy approaches physicians at ≥ 4000 cases” (numeric NR); EM deviation 0.5
6	[23]	2020	Diagnosis and staging	MLP (multilayer NN)	> 8000	Accuracy; ROC analysis	System accuracy 61%→87% as *n* increases; experts 97%→81%; ROC operating point at (TPR = 1, FPR = 0); AUC NR
7	[24]	2022	Diagnosis (PCa/csPCa) in PSA gray zone	DNN; XGBoost	18,824	AUROC; comparison with PHI	DNN AUROC 0.743 (internal), 0.704 (external); > PHI (exact PHI AUROC NR)
8	[25]	2022	Workflow/dashboard CDSS (no predictive model)	NLP + FHIR‑based integration	10	Decision time; usability	Decision time −59.9% (191.44→76.72 s); usability Cohen's d 4.94
9	[26]	2023	HDR brachytherapy plan optimization	EA (MO‑RV‑GOMEA)	14	Dose‑volume; LCI/LSI; selection/approval time	Improved OAR trade‑offs; time ↓ (NR); LCI/LSI numeric NR
10	[27]	2023	Survival (CSM/OM) and CRPC treatment sequencing	XGBoost (best)	801	C‑index (by line of therapy)	CSM: first 0.827, second 0.807, third 0.748; OM: first 0.822, second 0.813, third 0.729

*Note*: change from baseline to optimized; ↓/↑ decrease/increase; NR not reported; κ Cohen's kappa. Abbreviations. Acc accuracy; AUROC area under ROC curve; BCR, biochemical recurrence; CDSS, clinical decision support system; CI, confidence interval; C‐index, Harrell's concordance index; CSPC, clinically significant prostate cancer; CSM/OM, cancer‐specific/overall mortality; CRPC, castration‐resistant prostate cancer; Dx, diagnosis; DNN, deep neural network; EA, evolutionary algorithm; ELR, exponential linear regression (ensemble); EM, evaluation of malignancy (study‐specific); FHIR, fast healthcare interoperability resources; GA, genetic algorithm; HDR, high‐dose‐rate; IMRT, intensity‐modulated radiotherapy; LCI/LSI, least coverage/sparing indices; LSTM, long short‐term memory; MCC, Matthews correlation coefficient; MO‐RV‐GOMEA, multi‐objective real‐valued gene‐pool optimal mixing evolutionary algorithm; MLP/MLP‐N, multilayer perceptron / N‐year variant; mp‑MRI, multiparametric MRI; NN, neural network; NPV/PPV, negative/positive predictive value; TPR/FPR, true‐positive/false‐positive rate; OAR, organ at risk; PCa, prostate cancer; PFS, progression‐free survival; PHI, prostate health index; ProKnow, plan‑quality scoring framework; ROC, receiver operating characteristic; RBF, radial basis function kernel; RL, reinforcement learning; Sens/Spec, sensitivity/specificity; SVM, support vector machine; Tx, treatment; VTPN, virtual treatment planner network.

As summarized in Table 2, performance across AI‐based CDSSs varied according to the target task and model family. Neural‐network‐based architectures (e.g., LSTM, MLP, and DNN) consistently outperformed traditional regression baselines for survival and recurrence prediction, achieving C‐indices of up to 0.92 and AUROC values around 0.84. Gradient‐boosting models demonstrated competitive discrimination in recurrence prediction (AUROC ≈ 0.84), whereas reinforcement‐learning and EAs markedly improved radiotherapy plan quality and reduced optimization time. Diagnostic systems that combined mp‐MRI or multimodal data achieved accuracies between 0.87 and 0.89 with κ ≈ 0.80, approaching expert‐level interpretation. In contrast, workflow‐oriented CDSSs that relied on natural‐language processing and FHIR integration mainly improved operational outcomes, shortening decision‐making time by nearly 60%. Collectively, these findings indicate that while deep and ensemble models deliver the highest predictive accuracy, practical integration and validation remain limited, emphasizing the need for standardized evaluation across tasks.

### 4.3 Addressing Research Question 3

Alignment with CDSS Standards: Three key areas were evaluated: UI and interaction, data entry techniques, and integration with healthcare systems. UI and Interaction: Studies [18, 19, 20, 24, 26] used UI for data entry and visualization, though study [26] employed a special workflow for treatment planning software; these investigations most likely included manual data entry. Although studies [27] and [22] did not specifically mention a UI, their web‐based format and emphasis on clinical implementation [27] or prospective UI integration [22] suggested the possibility of user involvement. It's interesting to note that study [21] used a stand‐alone application with a GUI, but user participation was restricted to plan selection based on pre‐established score rather than real‐time decision support. Study [25] also used a web‐based UI that centralized clinical, imaging, and pathology data into a single dashboard. Finally, study [23], did not specify the UI interaction, indicating that offline data analysis would be preferable to real‐time clinical integration. Integration with EHR and information systems: The Barten's et al. [26] study, successfully integrated hospital information systems using DICOM for data exchange with Oncentra Brachy software for treatment planning. Another noteworthy exception was made by Henkel et al. [25], which integrated directly with LIS, RIS, PACS, and pathology systems via FHIR, enabling bidirectional data flow and structured reporting back into the EHR. Furthermore, study [18] suggested future RAPIDMINER, an open‐source program, integration with EHR. Similarly, study [21] mentioned potential API interaction with treatment planning systems in the future. Data Input Methods: Studies [18, 19, 20, 22, 24, 26] likely involved manual data entry through UIs. study [24] used in‐model imputation to handle missing data points. Studies lacking UI information [21, 23, 27] may require manual data preparation or unspecified integrations. Henkel et al. [25] automated the entire data aggregation process, requiring manual replacement only for externally scanned papers. Adherence to CDSS Standards: While none of the studies explicitly mentioned following specific CDSS design standards, the functionalities described in some (e.g., user interaction, result visualization, recommendations) aligned with general CDSS principles. The system developed by Henkel et al. [25] was one of the few to explicitly adopt HL7 FHIR standards, SNOMED CT, and LOINC.

### 4.4 Addressing Research Question 4

Clinical decision‐making phases: We classified the studies based on their involvement in the key clinical phases: prediction, diagnosis, and treatment management.

Predicting patient outcomes was a major strategy used by a considerable number of studies [18, 19, 20, 21, 24, 27] to support severity evaluation. The goal of studies [19] and [20] was to estimate the probability of BCR, facilitate prognosis talks, and maybe have an impact on treatment choices. Study [27] predicted survival outcomes for the several treatment choices for CRPC, enabling physicians to select the most appropriate therapy based on severity of the disease. Studies [18] and [24] provided an assessment of the likelihood and severity of cancer, assisting medical professionals in deciding how serious the condition is and what to do next. Interestingly, study [21] enhanced radiation therapy planning by evaluating the impact of radiation on the tumor and surrounding tissues, which helped improve the assessment of severity. Combined diagnosis and management: A smaller set of trials [22, 26] dealt with both the disease's diagnosis and possible courses of treatment. By identifying treatment‐required locations and subsequently optimizing treatment strategies for efficient disease control, study [26] indirectly aided in diagnosis. Study [22], on the other hand, provided a more thorough methodology. Tumor classification, SVM‐based cancer staging, stage‐based treatment plan recommendations, and therapy efficacy monitoring were all included. Study [23], meanwhile, focused solely on diagnosis, without assessing the disease's severity or management. Similarly, Henkel et al. [25], supported both diagnosis and treatment recommendation through guideline‐based suggestions, though it lacked predictive or monitoring features. Overall, the studies provide strong evidence of CDSS effectiveness in aiding clinical decision‐making in PCa, with some studies excelling in predicting and others offering a more comprehensive approach that included diagnosis, treatment recommendations, and even therapy monitoring.

## 5 Discussion

This study examined AI‐based CDSS in PCa, focusing on data quality, technical aspects, user interaction, integration with healthcare information systems, and adherence to CDSS standards. It offers insights into the current landscape and potential future impact of AI‐powered CDSS in transforming PCa management. The study also underlines limitations and suggests directions for future research to ensure the responsible and effective integration of AI in PCa care.

**FIGURE 3:**
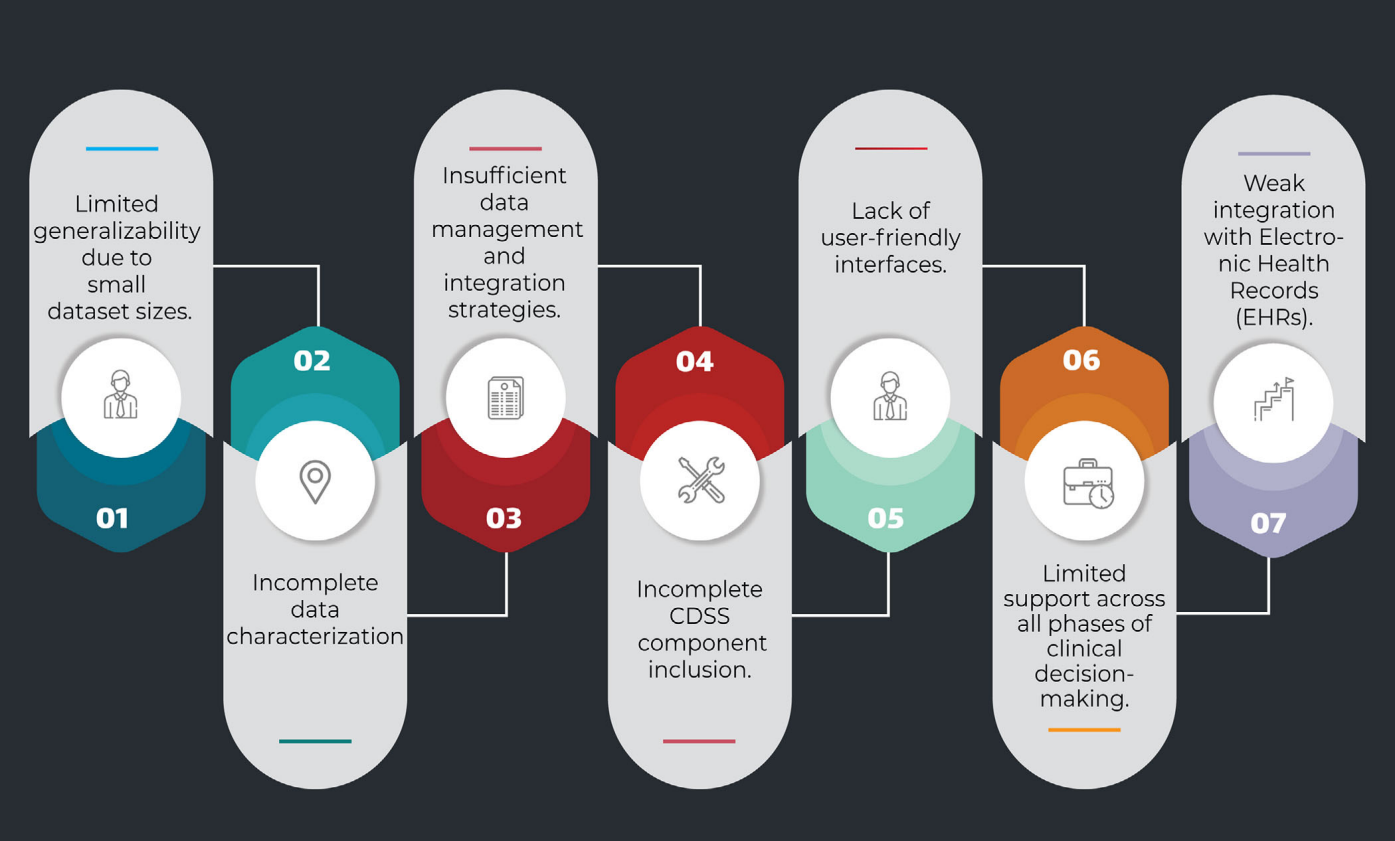
Limitations reported across the included studies.

Figure 3 summarizes the limitations reported across the included studies.

In the following section, we will discuss the limitations based on Research Questions.

Sample size and generalizability: While there is no definitive sample size for training AI models, existing literature suggests that the complexity of the model and the required accuracy level will determine how much data is required [28, 29]. It may be adequate to use thousands of data points for basic tasks [28]. Millions of records, however, may be needed for more complex models [29]. While larger datasets [19, 20, 22, 23, 24] may improve generalizability, they often introduce challenges related to data quality and consistency. For example, a study with over 18,000 cases supports more robust model training [30], while smaller cohorts can lead to less reliable models [31].

Data characterization: AI has shown potential in enhancing diagnostic accuracy, improving prognosis prediction, refining therapy planning, and staging [32]. In Diagnosis, AI models integrate clinical and imaging data, notably multiparametric MRI (mp‐MRI) for detailed anatomical insights [33]. AI models also use clinical data, such as some tumor markers like PSA, prostate‐specific membrane antigen (PSMA) and free PSA (fPSA) [34], alongside patient demographics of age, ethnicity, biopsy results and Gleason score, to enhance diagnostic accuracy [33]. However, some studies [18, 22] and [23] used limited data to train these models. Study [22] focuses only on six tumor markers (PSA, PSMA, red blood cell (RBC), hemoglobin (HB), prostatic acid phosphatase (PAP)), and study [23], combines the same tumor markers with medical imaging data but still lacks other information such as medical records or cancerous region information. Additional biomarkers like Human Glandular Kallikrein 2 (hK2) [35] and urokinase plasminogen activator (uPA) [36] have proven to be useful in diagnostics, but were not included in these studies. In prognosis, AI models predict cancer progression, recurrence, and survival by analyzing clinical and imaging data [37], and also assess gene expression patterns linked to aggressive PCa [37, 38]. However, studies [19, 20, 27] and [24] face limitations in data range. Studies [19] and [20] lack details on tumor region and treatment history, study [24] does not complete BMI statistics, total prostate volume (TPV) for certain patients, and free PSA values (fPSA) which are crucial for prognosis. Comprehensive data integration remains essential for accurate predictions [39]. Regarding treatment plans, the data used for diagnosis and prognosis can be helpful [40]. Study [26] uses MRI scans to detect target volumes but lacks treatment history, limiting its ability to adjust treatment regimens. Similarly, study [21] focuses on treatment plans but does not incorporate any imaging data, PSA levels, clinical data, or patient demographics. Hence, in general, treatment history is essential to refine approaches for specific patient needs [41, 42, 43]. For staging, AI relies heavily on mp‐MRI images to access tumor size, location, and capsular involvement [44]. In addition to imaging data, AI models combine Gleason scores [45, 46], PSA levels [47], and results from the digital rectal exam (DRE) [48]. Advanced cancer staging may be indicated by elevated Gleason scores, aberrant DRE findings, and increased PSA values [46, 47, 48]. However, study [22] only uses six tumor markers to train the staging model, which is similar to the limitations on treatment planning covered in Study [21].

AI models: AI has significantly impacted cancer care, especially for diagnosis, treatment planning, and prognosis [49, 50, 51]. Among the latest AI techniques, CNNs outperform in analyzing medical images for tumor detection and staging [52]. SVMs assist in feature selection and classification [53], while random forests enhance prediction accuracy [54]. AI also uses omics data to identify biomarkers and personalize therapies [55]. Several studies [19, 21, 24, 27] applied models such as LSTM, XGBoost, DNN, SVM. While LSTM offers better interpretability [56], models like DNN and SVM can be less interpretable due to their complexity [57], which reduces healthcare providers trust in the models' output, including diagnosis [58], treatment recommendation [59], or risk prediction [60]. Newer models like classification trees and graph transformer networks show improved interpretability [61, 62]. The performance of these models depends on the quality and quantity of training data. Most of the included studies did not mention a data validation process. Aside from the study using EHR data [26], others relied on datasets prone to biases from manual collection, limiting model generalizability and accuracy [63]. Additionally, XGBoost, and DNN models [24, 27] face the risk of overfitting, especially with small datasets [64]. Some models [21, 27] demand significant computational resources, which may be impractical in resource‐limited healthcare settings [65].

CDSS structure: CDSSs aim to improve healthcare quality by supporting physicians decision‐making [66]. The essential components of AI‐driven CDSS architecture include a knowledge base, inference engine, data management layer, and UI [9, 12, 13].

AI‐driven CDSSs, especially those using deep learning, can uncover patterns in large datasets that traditional methods may miss [63]. Integrating real‐time data from EHRs into these systems enables ongoing learning and adaption to new knowledge [10]. EHR integration improves decision‐making, treatment ordering, and preventative care [67, 68]. In cancer care, integrating EHR records into CDSSs has led to significant improvements in prediction accuracy, cost reduction, enhanced clinical workflow, and guideline adherence [69, 70]. However, Barten et al.'s study [26] is the only one to successfully integrate with hospital information systems via DICOM. The efficacy and user acceptance of AI‐based CDSSs depend heavily on their UI, which should be intuitive, support clinician workflows, and present data clearly [13, 71]. Visualization tools, like graphs and heat maps, help improve decision accuracy, reduce cognitive load, and ultimately improve patient outcomes [72, 73, 74, 75].

The reviewed studies on AI‐powered systems in PCa care show varied adherence to core CDSS components. While all studies utilize AI models, only some studies [22, 24, 27] mentioned using clinical data or guidelines to inform the AI models, suggesting a form of knowledge base. Studies on diagnosis using SVM [18], virtual treatment planning with DRL [21], and diagnosis using a perceptron neural network [23] don't explicitly reference elements like the knowledge base. Data management and UI aspects are also inconsistently addressed. Studies on CRPC [27], risk stratification [24], and multi‐stage CDSS [22] describe data collection (like EHR, demographics) and UIs for data input and result visualization. In contrast, studies [18, 21, 23] don't mention these components. None of the studies explicitly mention adherence to specific CDSS design standards. Recent studies emphasize the importance of core CDSS components, such as the knowledge base, inference engine, data management layer, and UI. This trend is evident in research on colorectal cancer [76], breast cancer [77, 78], and other malignancies [70], enhancing diagnostic and treatment accuracy.

Real‐world applicability of AI‐CDSS: AI‐based CDSS in PCa can improve decision‐making by assisting physicians in accurately diagnosing and staging the disease. However, real‐world implementation faces challenges such as clinician acceptance, integration with EHR systems, and the need for high‐quality data. To achieve widespread adoption, AI‐based CDSS must demonstrate clinical efficacy, be cost‐effective, and integrate smoothly into existing health care workflows. Despite the promise of AI‐CDSS, real‐world integration will depend on addressing concerns such as clinician trust in AI recommendations, the need for continuous training on evolving datasets, and interoperability with existing medical systems, particularly EHRs and clinical databases. Moreover, AI models should be evaluated in clinical trials to establish their effectiveness in improving patient outcomes and optimizing treatment decisions [79]. The study [25] demonstrated that AI‐powered CDSS can significantly enhance workflow efficiency by automating data integration from multiple hospital systems, reducing clinical decision‐making time by 60%, especially in complex, multi‐source datasets.

Decision‐making phases: A well‐designed CDSS should ideally integrate all three main phases of the decision‐making process [9]. During diagnosis, CDSS utilizes its knowledge base of symptoms, diseases, and patient data to suggest differential diagnoses and relevant tests [10, 80]. In severity assessment, the system can analyse clinical guidelines, demographics, and laboratory results to calculate severity scores or identify potential complications [80, 81]. During management, CDSS provides evidence‐based treatment recommendations or tailored order sets based on the condition's severity [82]. However, not all CDSSs offer this comprehensive functionality. Some focus only on one phase, like diagnosis (suggesting differential diagnoses) or severity assessment (risk factor analysis), while all phases are crucial for effective care. Many of the included studies [18, 19, 20, 21, 24, 27] concentrate on severity assessment, predicting outcomes like BCR or overall survival. Fewer studies [22, 26] tackle both diagnosis and management. One study [26] indirectly aids diagnosis by identifying treatment targets but doesn't suggest a diagnosis. Another study [22] covers tumor classification, staging, treatment recommendations, and therapy monitoring. However, the limited number of studies exploring this combined approach highlights a gap in CDSS development. Study [23] focuses solely on diagnosis without assessing severity or treatment options.

### 5.1 Suggestions for Further Research and Improvement

Future research should employ more varied datasets that incorporate a greater variety of clinical characteristics, such as tumor markers and patient demographics, in order to increase generalizability. To guarantee consistent and trustworthy input, strong data management and integration techniques are necessary. To facilitate clinical use, CDSS designs should combine essential elements like an inference engine, knowledge base, and user‐friendly interface with features like interactive tools and real‐time data visualization. Improving the quality of care also requires real‐time EHR integration. Despite the encouraging accuracy of several research, few have achieved clinical validation or shown practical impact. In the future, studies should concentrate on assessing AI‐CDSS in actual clinical settings, stressing cost‐effectiveness, workflow compatibility, and user confidence.

### 5.2 Limitations of the Study

It is important to recognize the limitations of this systematic review. First, even with a thorough search across several databases and additional manual searches, it is still possible that pertinent research was overlooked, especially since we were limited to English‐language publications. Second, a meta‐analysis was not possible due to the included research' variability or missing data in terms of sample sizes, AI models, data inputs, and study designs, which also made it difficult for us to make quantitative comparisons or reach firm conclusions. Lastly, subjectivity in data extraction and interpretation may have affected our findings even though efforts were made to minimize bias through independent review and to rigorously assess research quality.

## 6 Conclusion

Future studies can improve the efficacy, dependability, and usefulness of AI‐based CDSS in PCa management, from diagnosis to treatment and care, by addressing these limitations. Additionally, clinicians are advised to critically evaluate AI‐CDSS tools before applying them in clinical workflows while awaiting stronger validation from large‐scale clinical trials.

## Author Contributions

**Senobar Naderian**: conceptualization, methodology, writing original draft, screening, data extraction. **Farzin Soleimanzadeh**: conceptualization, validation, supervision and review. **Leila Nikniaz**: methodology, data curation, validation. **Sarvin Sanaie**: validation. **Fatemeh Sadeghi‐Ghyassi**: methodology, data curation. **Taha Samad‐Soltani**: conceptualization, supervision and review.

## Funding

The authors have nothing to report.

## Ethics Statement

This systematic review does not involve new human participants. All data included in this review were derived from publicly available studies that were approved by appropriate ethical committees.

## Consent

The authors have nothing to report.

## Conflicts of Interest

The authors declare no conflicts of interest.

## Supporting information




**Supplementary File 1**: htl270026‐sup‐0001‐SuppMat.docx

**Supplementary File 2**: htl270026‐sup‐0001‐SuppMat.docx

**Supplementary File 3**: htl270026‐sup‐0001‐SuppMat.docx

## Data Availability

All data supporting the findings of this study are available within the article and its supplementary material.
