# Supplementary material for: A Systematic Review of Artificial Intelligence‐Based Clinical Decision Support Systems in Prostate Cancer Management
Source: Healthc Technol Lett. 2025 Nov 18;12(1):e70026. doi: 10.1049/htl2.70026 (PMC12625777; doi:10.1049/htl2.70026)
Supplement: Supplementary file 1 — htl270026‐sup‐0001‐SuppMat.docx [file HTL2-12-e70026-s003.docx]

**Supplementary File 1
The search strategy employed across databases:**

- 1. Keywords related to Artificial Intelligence (AI) such as: "Artificial Intelligence" , "Machine Learning" , "Deep Learning" , "Supervised Machine Learning" , "Unsupervised Machine Learning" , "Semi supervised learning" , "Reinforcement learning" , "Automated" , "Semi-automated" , "Neural Networks
  2. Terms associated with Prostate Cancer/Neoplasm including Prostate Cancer/Neoplasm, including "Prostatic Neoplasms", "Prostate Cancer", "Prostate Neoplasm", "Prostatic Adenocarcinoma", "Prostate Adenocarcinoma", "Prostate Tumor", "Prostatic Cancer", "Prostatic Tumor".
  3. Concepts relevant to Clinical Decision Support Systems (CDSS) such as: "CDSS", "DSS", "AI-based CDSS", "Machine Learning-based CDSS", "Decision Support System", "Decision Support Software", "Decision Support Technology", "Decision Support Framework", "Decision Support tool", "Decision Support Application", "Decision Support Algorithm", "Decision Support Implement".
