# Supplementary material for: A Systematic Review of Artificial Intelligence‐Based Clinical Decision Support Systems in Prostate Cancer Management
Source: Healthc Technol Lett. 2025 Nov 18;12(1):e70026. doi: 10.1049/htl2.70026 (PMC12625777; doi:10.1049/htl2.70026)
Supplement: Supplementary file 2 — htl270026‐sup‐0001‐SuppMat.docx [file HTL2-12-e70026-s001.docx]

**Supplementary File 1**

Pubmed full search strategy

| 1 | Artificial Intelligence | "Artificial Intelligence"[MeSH Terms] OR "Artificial Intelligence"[Title/Abstract] OR "Machine Learning"[MeSH Terms] OR "Machine Learning"[Title/Abstract] OR "Deep Learning"[Mesh Terms] OR "Deep Learning"[Title/Abstract] OR "Supervised Machine Learning"[MeSH Terms] OR "Supervised Machine Learning"[Title/Abstract] OR "Unsupervised Machine Learning"[MeSH Terms] OR "Unsupervised Machine Learning"[Title/Abstract] OR "Semi supervised learning"[Title/Abstract] OR "Reinforcement learning"[Title/Abstract] OR "Automated"[Title/Abstract] OR "Semi-automated"[Title/Abstract] OR "neural networks, computer"[MeSH Terms] OR "Neural Networks"[Title/Abstract] |  |
| --- | --- | --- | --- |
| 2 | Prostate Cancer/ Neoplasm | "Prostatic Neoplasms"[MeSH Terms] OR "Prostatic Neoplasm*"[Title/Abstract] OR "Prostate Cancer*"[Title/Abstract] OR "Prostate Neoplasm*"[Title/Abstract] OR "prostatic adenocarcinoma"[Title/Abstract] OR "prostatic cancer*"[Title/Abstract] OR "prostate adenocarcinoma"[Title/Abstract] OR "prostate tumor*"[Title/Abstract] OR "prostatic tumor*"[Title/Abstract] OR "cancer of prostate"[Title/Abstract] OR "tumor of prostate"[Title/Abstract] |  |
| 3 | CDSS | "decision support systems, clinical"[MeSH Terms] OR "Decision Support Techniques"[MeSH Terms] OR "decision making, computer assisted"[MeSH Terms] OR "diagnosis, computer assisted"[MeSH Terms] OR "decision support systems, management"[MeSH Terms] OR "CDSS"[Title/Abstract] OR "DSS"[Title/Abstract] OR "AI based CDSS"[Title/Abstract] OR "machine learning-based CDSS"[Title/Abstract] OR "decision support system*"[Title/Abstract] OR "Decision Support Software"[Title/Abstract] OR "decision support technolog*"[Title/Abstract] OR "decision support framework*"[Title/Abstract] OR "decision support tool*"[Title/Abstract] OR "Decision Support Application"[Title/Abstract] OR "Decision Support Algorithm"[Title/Abstract] OR "decision support implement*"[Title/Abstract] OR "clinical decision support system*"[Title/Abstract] OR "medical decision support system*"[Title/Abstract] OR "Decision Aid"[Title/Abstract] OR "decision algorithm*"[Title/Abstract] OR "real time clinical decision support system*"[Title/Abstract] OR "CDSS"[Title/Abstract] OR "DSS"[Title/Abstract] OR "computerized decision support system*"[Title/Abstract] OR "Computerized Decision Support Software"[Title/Abstract] OR "computerized decision support technolog*"[Title/Abstract] OR "computerized decision support tool*"[Title/Abstract] OR "interactive decision support system*"[Title/Abstract] OR "interactive decision support tool*"[Title/Abstract] |  |
| 1 AND 2 AND 3 | | ("Artificial Intelligence"[MeSH Terms] OR "Artificial Intelligence"[Title/Abstract] OR "Machine Learning"[MeSH Terms] OR "Machine Learning"[Title/Abstract] OR "Deep Learning"[MeSH Terms] OR "Deep Learning"[Title/Abstract] OR "Supervised Machine Learning"[MeSH Terms] OR "Supervised Machine Learning"[Title/Abstract] OR "Unsupervised Machine Learning"[MeSH Terms] OR "Unsupervised Machine Learning"[Title/Abstract] OR "Semi supervised learning"[Title/Abstract] OR "Reinforcement learning"[Title/Abstract] OR "Automated"[Title/Abstract] OR "Semi-automated"[Title/Abstract] OR "neural networks, computer"[MeSH Terms] OR "Neural Networks"[Title/Abstract]) AND ("Prostatic Neoplasms"[MeSH Terms] OR "prostatic neoplasm*"[Title/Abstract] OR "prostate cancer*"[Title/Abstract] OR "prostate neoplasm*"[Title/Abstract] OR "prostatic adenocarcinoma"[Title/Abstract] OR "prostatic cancer*"[Title/Abstract] OR "prostate adenocarcinoma"[Title/Abstract] OR "prostate tumor*"[Title/Abstract] OR "prostatic tumor*"[Title/Abstract] OR "cancer of prostate"[Title/Abstract] OR "tumor of prostate"[Title/Abstract]) AND ("decision support systems, clinical"[MeSH Terms] OR "Decision Support Techniques"[MeSH Terms] OR "decision making, computer assisted"[MeSH Terms] OR "diagnosis, computer assisted"[MeSH Terms] OR "decision support systems, management"[MeSH Terms] OR "CDSS"[Title/Abstract] OR "DSS"[Title/Abstract] OR "AI based CDSS"[Title/Abstract] OR "machine learning-based CDSS"[Title/Abstract] OR "decision support system*"[Title/Abstract] OR "Decision Support Software"[Title/Abstract] OR "decision support technolog*"[Title/Abstract] OR "decision support framework*"[Title/Abstract] OR "decision support tool*"[Title/Abstract] OR "Decision Support Application"[Title/Abstract] OR "Decision Support Algorithm"[Title/Abstract] OR "decision support implement*"[Title/Abstract] OR "clinical decision support system*"[Title/Abstract] OR "medical decision support system*"[Title/Abstract] OR "Decision Aid"[Title/Abstract] OR "decision algorithm*"[Title/Abstract] OR "real time clinical decision support system*"[Title/Abstract] OR "CDSS"[Title/Abstract] OR "DSS"[Title/Abstract] OR "computerized decision support system*"[Title/Abstract] OR "Computerized Decision Support Software"[Title/Abstract] OR "computerized decision support technolog*"[Title/Abstract] OR "computerized decision support tool*"[Title/Abstract] OR "interactive decision support system*"[Title/Abstract] OR "interactive decision support tool*"[Title/Abstract]) | 771 |
